# Supplementary material for: An ultra-high gain single-photon transistor in the microwave regime
Source: Nat Commun. 2022 Oct 15;13:6104. doi: 10.1038/s41467-022-33921-6 (PMC9569345; doi:10.1038/s41467-022-33921-6)
Supplement: Supplementary file 1 — Supplementary Information [file 41467_2022_33921_MOESM1_ESM.pdf]

Supplementary Information

Zhiling Wang[1,*] Zenghui Bao[1,*] Yan Li[1,*] Yukai Wu[1,2], Weizhou Cai[1], Weiting Wang[1], Xiyue Han[1], Jiahui Wang[1], Yipu Song[1,2], Luyan Sun[1,2], Hongyi Zhang[1,2,†] and Luming Duan[1,2‡]
[1]*Center for Quantum Information, Institute for Interdisciplinary Information Sciences, Tsinghua University, Beijing 100084, PR China and*
[2]*Hefei National Laboratory, Hefei 230088, PR China*
(Dated: October 1, 2022)

CONTENTS

SUPPLEMENTARY NOTE 1 - Sample information and experiment setup 2

SUPPLEMENTARY NOTE 2 - Photon number calibration 2

SUPPLEMENTARY NOTE 3 - Calibrate and optimize the photon-gating process 4

SUPPLEMENTARY NOTE 4 - Quantum state tomography on reflected gate photons 5

SUPPLEMENTARY NOTE 5 - Error analysis for gate-photon tomography 6

SUPPLEMENTARY NOTE 6 - Theoretical model for photon switching 8
A. Gate by an ideal single-photon source 8
B. Gate by weak coherent state photons 9
C. Error estimation for the photon-gating process 10

SUPPLEMENTARY NOTE 7 - Varied signal pulse length 11

SUPPLEMENTARY NOTE 8 - Cavity transmission in the presence of a strong input signal 12

Supplementary References 13

* These authors contributed equally.
† hyzhang2016@tsinghua.edu.cn
‡ lmduan@tsinghua.edu.cn

SUPPLEMENTARY NOTE 1 - SAMPLE INFORMATION AND EXPERIMENT SETUP

As described in the main text, the single-photon transistor contains two 3D microwave cavities. Cavity I is used for the detection of the gate photons. The presence of a single gate photon would change the quantum state of the superconducting qubit coupled to both of the two cavities. Cavity II is used to switch the incoming signal photons according to the state of the qubit. In Supplementary Table 1 we list the detailed information of the device. Here the frequency of cavity I, $\omega_{\mathrm{I}}^{\mathrm{c}} = (\omega_{\mathrm{I}}^{\mathrm{c},|\mathrm{g}\rangle} + \omega_{\mathrm{I}}^{\mathrm{c},|\mathrm{e}\rangle})/2$, stands for the average of resonance frequencies for cavity I when the qubit is in $|\mathrm{g}\rangle$ and $|\mathrm{e}\rangle$. It is also the frequency of the gate photon, which is marked as the dashed purple line in Fig. 1(f) of the main text.

Supplementary Table 1. System parameters

cavity I frequency, $\omega_{\mathrm{I}}^{\mathrm{c}}/2\pi$(GHz)	8.00398
cavity I internal loss rate, $\kappa_{\mathrm{I}}^{\mathrm{i}}/2\pi$(MHz)	0.19
cavity I out-coupling rate, $\kappa_{\mathrm{I}}^{\mathrm{r}}/2\pi$(MHz)	1.81
cavity II resonance frequency for qubit in $|\mathrm{e}\rangle$, $\omega_{\mathrm{II}}^{\mathrm{c},|\mathrm{e}\rangle}/2\pi$(GHz)	7.24595
cavity II bare frequency, $\omega_{\mathrm{II}}^{\mathrm{c,bare}}/2\pi$(GHz)	7.23795
cavity II internal loss rate, $\kappa_{\mathrm{II}}^{\mathrm{i}}/2\pi$(MHz)	0.04
cavity II out-coupling rate, $\kappa_{\mathrm{II}}^{\mathrm{in/out}}/2\pi$(MHz)	0.13
qubit frequency, $\omega_{\mathrm{q}}/2\pi$(GHz)	5.34996
qubit anharmonicity, $E_{\mathrm{c}}/2\pi$(MHz)	248.73
dispersive coupling rate for cavity I, $\chi_{\mathrm{I}}^{\mathrm{ge}}/2\pi$(MHz)	-0.865
dispersive coupling rate for cavity II, $\chi_{\mathrm{II}}^{\mathrm{ge}}/2\pi$(MHz)	-0.947
dispersive coupling rate for cavity II, $\chi_{\mathrm{II}}^{\mathrm{gf}}/2\pi$(MHz)	-1.759
qubit energy relaxation time of $|\mathrm{e}\rangle$, $T_1^{\mathrm{ge}}(\mu s)$	40
qubit dephasing time of $|\mathrm{e}\rangle$, $T_2^{\mathrm{ge}}(\mu s)$	20
qubit energy relaxation time of $|\mathrm{f}\rangle$, $T_1^{\mathrm{ef}}(\mu s)$	31
qubit dephasing time of $|\mathrm{f}\rangle$, $T_2^{\mathrm{ef}}(\mu s)$	9.71

The measurement setup is schematically shown in Supplementary Figure 1. To generate a coherent pulse with desired amplitude and phase, continuous-wave gigahertz carrier signals are modulated by a megahertz signal with an IQ mixer. The megahertz signal is generated from an arbitrary waveform generator (AWG) and the gigahertz signal is generated with a microwave signal generator. In Supplementary Figure 1, the right (left) AWG and signal generator are used to generate the gate (signal) photon pulse. Both gate photon pulse and signal photon pulse are sent to the sample through multiple attenuators and filters to suppress the thermal noise. To measure the reflected gate photon signal, a circulator is used before the input port of cavity I.

The reflected gate photons are successively amplified by a high-electron-mobility transistor (HEMT) at 4-K plate and two room temperature RF amplifiers. For the signal photons, an addition Josephson parametric amplifier (JPA) is used at the mixing chamber stage to effectively detect weak signal pulses with less than 100 photons. The amplified signals for both signal photons and gate photons are acquired with a homodyne method. The amplified signals are demodulated with a mixer to an intermediate frequency (IF) of 50 MHz and are sent into the corresponding analog-to-digital converter (ADC) with 1 GHz sampling rate. These two ADCs are synchronized during the data acquisition, which enables us to match the measurement result for gate photons and that for signal photons in each of the experiment trials. The quadrature components of the measured signals can be obtained with a digital homodyne method. This setup enables us to perform quantum state tomography on the gate photons since it effectively realizes a homodyne measurement on the outputted photon field. Meanwhile, this setup also enables us to measure the transmission intensity of the signal photons. The transmission intensity in repeated measurements is further summarized as a histogram plot as illustrated in Supplementary Figure 2, which can be used to determine the working state of the transistor.

SUPPLEMENTARY NOTE 2 - PHOTON NUMBER CALIBRATION

The photon numbers of both the input signal and the gate photon pulse are important parameters for the characterization and calibration of our single-photon transistor. In the experiment, we first populate the cavity with certain photon flux. Since the photons in the cavity would introduce a dephasing term to the qubit state via AC stark effect, we could determine the cavity photon number by measuring the qubit dephasing with a Ramsey method.

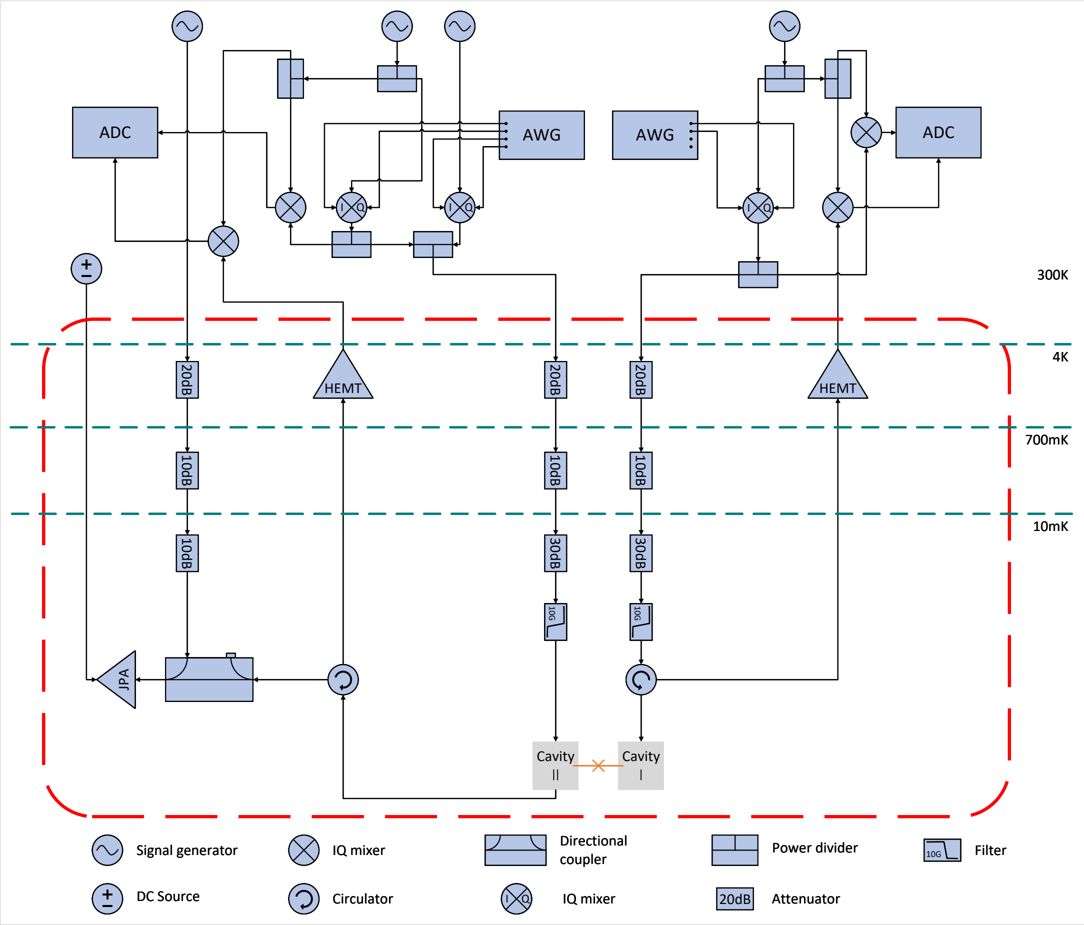

Supplementary Figure 1. An illustration of the experimental setup.

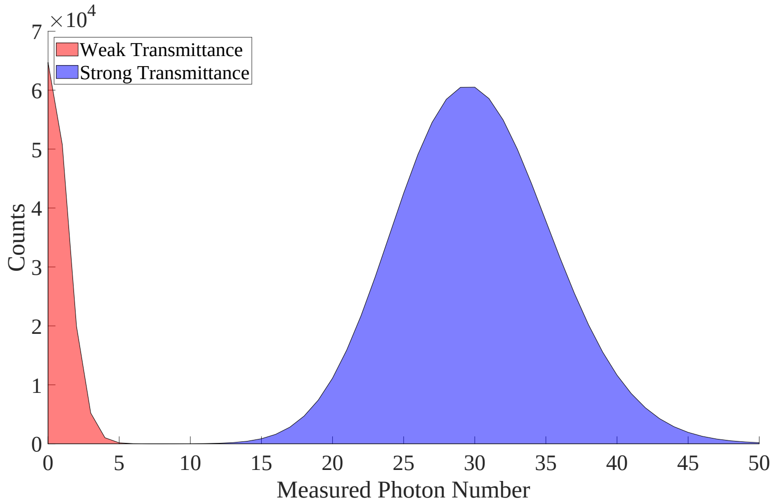

Supplementary Figure 2. Histogram plot of the measured transmission. Blue and red areas represent the strong and weak transmission, respectively, which corresponds to the ”on” and ”off” state of the transistor. The input signal photon number is $n_s = 37.2$. The threshold between two areas is given by the K-means clustering method. The bar plots shown in Fig. 2 in the main text show the sum of the red and blue areas of the histogram plot.

When a continuous coherent drive with photon flux $\dot{n}_{\rm d}$ at frequency $\omega_{\rm d}$ is sent to the cavity, the additional qubit

dephasing rate Γ_{m} would be [1]

$$\begin{aligned} \Gamma_{\mathrm{m}} &= \frac{\kappa_{\mathrm{tot}}\chi^2}{\kappa_{\mathrm{tot}}^2/4+\chi^2+\Delta_{\mathrm{d}}^2}(\bar{n}_+ + \bar{n}_-) \\ \bar{n}_\pm &= \frac{\kappa_{\mathrm{r}}\dot{n}_{\mathrm{d}}}{\kappa_{\mathrm{tot}}^2/4+(\Delta_{\mathrm{d}}\pm\chi)^2}, \end{aligned} \tag{1}$$

where $\bar{n}_\pm$ is the average photon number in the cavity when the qubit is in $|\mathrm{g}\rangle$ or $|\mathrm{e}\rangle$. $\Delta_{\mathrm{d}} = \omega_{\mathrm{d}} - \omega_{\mathrm{c}}$ is the detuning between the coherent driving and cavity frequency, κ_{r} is the decay rate of the input port for the photon flux, and κ_{tot} is the total decay rate of the cavity. In the experiment, we use the Ramsey method to determine the qubit dephasing rate with varied input signal strength. We observe a clear decrease in the qubit dephasing time constant when increasing the signal strength, as shown in Supplementary Figure 3.

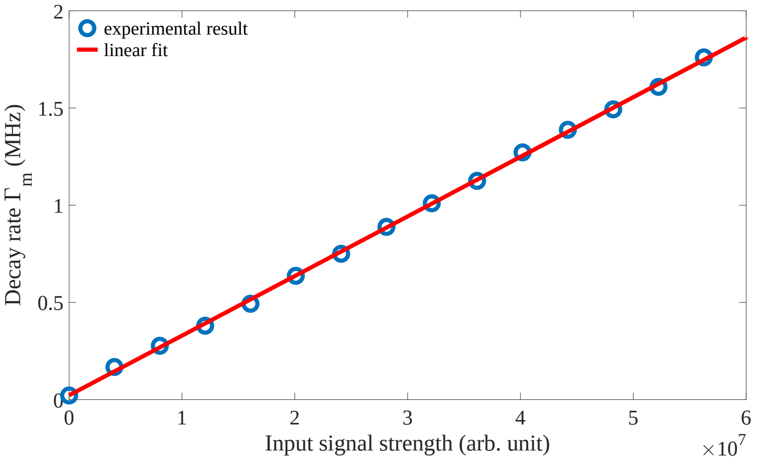

Supplementary Figure 3. Photon number calibration. The measured qubit dephasing rate as a function of the input signal strength. One could see the experimental qubit dephasing rate is linearly related to the input signal strength, which agrees well with the theoretical prediction. By comparing the experimental results and the theory, one could calibrate the experimental parameters to the absolute photon number.

By fitting the relation between the measured qubit dephasing rate and the applied input signal strength with Supplementary Equation 1, the corresponding photon flux can be extracted. Thus the total photon number n of the input pulse can be determined as

$$n = \int_0^{\mathrm{T}} \dot{n}_{\mathrm{d}}|f(t)|^2 dt. \tag{2}$$

Here T is the length of the input signal, and $f(t)$ is the temporal mode of the input signal. We used a Gaussian shape temporal mode $f(t)$ for the gate photon and a square pulse for the signal photon in the experiment. The width of the Gaussian shape is carefully chosen to achieve high photon-gating efficiency, as explained in the next section.

SUPPLEMENTARY NOTE 3 - CALIBRATE AND OPTIMIZE THE PHOTON-GATING PROCESS

We use the following scheme to calibrate the photon-gating efficiency. First, we apply a $\pi/2$ gate on the qubit, then send gate photons with a certain temporal mode to cavity I, and finally apply another $\pi/2$ gate with a π phase difference relative to the first qubit gate. Since the gate photons are in a weak coherent state, they could be well-approximated as either vacuum state $|0\rangle$ or Fock state $|1\rangle$ in each trial. The Fock state $|1\rangle$ of gate photon would introduce a π phase shift to the qubit and get the qubit in $|\mathrm{e}\rangle$ after the above-mentioned pulse sequence, whereas for the vacuum state of gate photon one would have the qubit in $|\mathrm{g}\rangle$. The measured statistical qubit population depends on the average photon number of the gate pulse. In the experiment, we measure the qubit state flip probability as a function of the gate photon number, which is fitted with a linear function [1]. The slop of the linear fit at the low-photon number regime reflects the qubit state flip probability induced by a single gate photon, or the gating efficiency

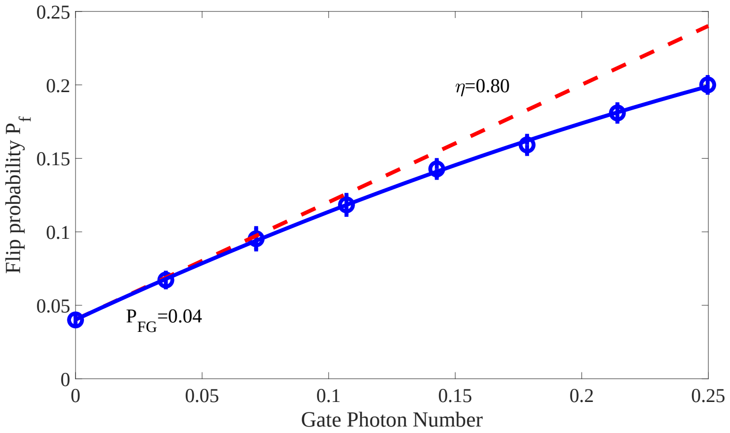

Supplementary Figure 4. Calibration of the single-photon gating efficiency. The measured qubit state flip probability as a function of the average gate photon number for a Gaussian-shaped coherent state gate photon pulse with $\sigma = 300$ ns and $T = 960$ ns. The red dashed line is a linear fitting result for the small-photon-number regime. The deviation between experimental data and the linear fit originates from the photon number statistics of the coherent state photons used in the experiment.

η. On the other hand, the intercept gives the probability that the qubit flips incorrectly when there is no gate photon, or false gating probability P_{FG}. An example that illustrates this calibration can be found in Supplementary Figure 4.

From the working principle of the single-photon transistor, the gate photons interact with the qubit through the resonance mode of cavity I. Because of the limited bandwidth of this cavity, the pulse shape of the gate photons could have a strong influence on the gating process. In the experiment, we use a Gaussian-shaped photon pulse to gate the transistor, which can be written as $f(t) = \exp\frac{-(t-T/2)^2}{2\sigma^2}$, where T is the total length of the gate photon and σ presents the width of the signal in the time domain. Intuitively, it is preferred to have the total gate time T as short as possible to mitigate the qubit decay and dephasing induced error. On the other hand, a larger σ is preferred to effectively fit the gate photons into the bandwidth of cavity I.

To optimize the photon-gating process, we calibrate the width σ and length T of the gate photon pulse by measuring gating efficiency η and false gating probability P_{FG}. We first applied gate photons with different lengths T but with a fixed $\sigma = 250$ ns. As shown in Supplementary Figure 5a, the gating efficiency reaches to the maximum value with $T/\sigma = 3.2$. We then scan the width of gate photons with a fixed T/σ ratio, as shown in Supplementary Figure 5c. Finally, we choose a Gaussian pulse with $\sigma = 300$ ns and $T = 960$ ns to envelop the gate photons, which leads to a single photon-gating efficiency of $\eta = 0.80$ and a false gating probability of $P_{\mathrm{FG}} = 0.04$. The corresponding experimental result is shown in Supplementary Figure 4.

SUPPLEMENTARY NOTE 4 - QUANTUM STATE TOMOGRAPHY ON REFLECTED GATE PHOTONS

As shown in Supplementary Figure 1, the reflected gate photons are measured with a homodyne setup [2, 3]. The two quadrature components measured in the experiment, I and Q, form the complex amplitude $S = I + iQ$ of the amplified photon field. Intuitively, this complex amplitude contains both the reflected photon modes and the noise added through the whole detection chain. Thus S can be written as $\hat{S} = \hat{a} + \hat{h}^\dagger$, where $\hat{a}$ is the annihilation operator of the propagating photon mode and $\hat{h}^\dagger$ is the creation operator of the noise mode. One of the main tasks of quantum state tomography is thus to recover the information related to the photon mode from the measured noisy complex amplitude.

Assuming that the added noise does not correlate with the signal, the moment term $\langle (\hat{S}^\dagger)^m \hat{S}^n \rangle$ of measured complex amplitude S can be written as

$$\langle (\hat{S}^\dagger)^m \hat{S}^n \rangle = \sum_{i,j=0}^{m,n} \binom{n}{j} \binom{m}{i} \langle (\hat{a}^\dagger)^i \hat{a}^j \rangle \langle \hat{h}^{m-i} (\hat{h}^\dagger)^{n-j} \rangle. \quad (3)$$

We also measure the complex amplitude when the input photon state is prepared as vacuum state, which yields $\hat{S}_{\mathrm{vac}} = \hat{h}^\dagger$. Thus $\langle \hat{h}^{m-i} (\hat{h}^\dagger)^{n-j} \rangle$ can be obtained from $\langle (\hat{S}^\dagger_{\mathrm{vac}})^{m-i} \hat{S}^{n-j}_{\mathrm{vac}} \rangle$. By solving these equations, the moments of

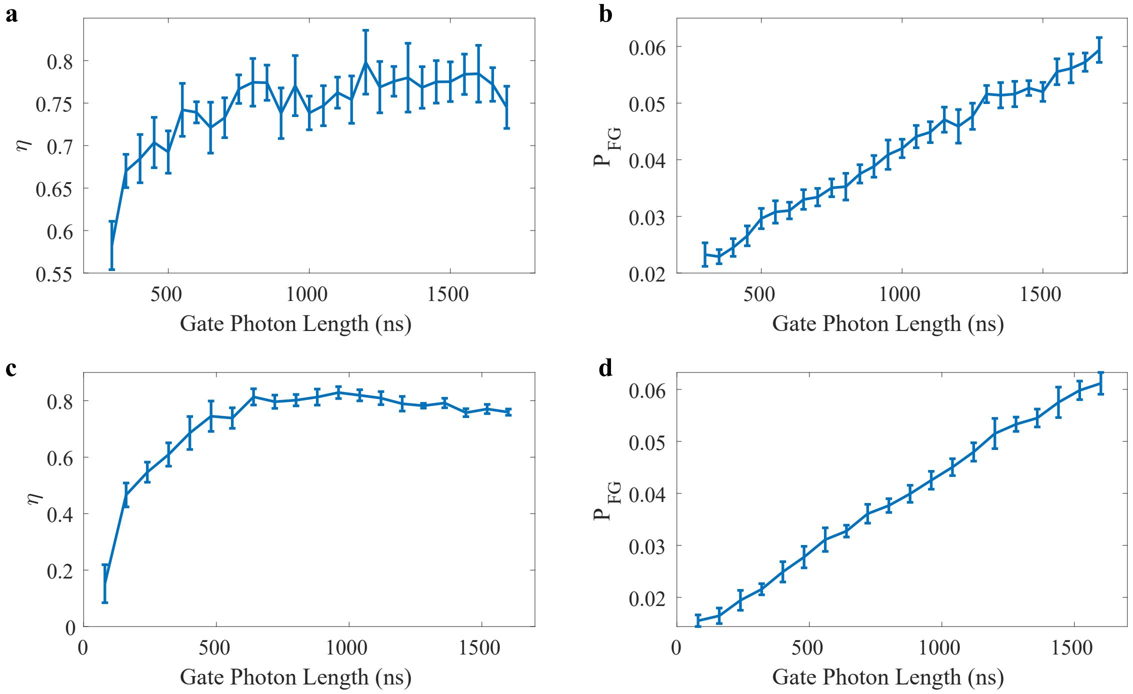

Supplementary Figure 5. Calibration of the photon-gating efficiency with different pulse shape. **a** and **b** show the calibrated photon-gating efficiency η and false gating probability P_{FG}, respectively, with a fixed $\sigma = 250$ ns but varied pulse length T. We find a maximized gating efficiency η, at the same time keeping the false gating probability as low as possible, which corresponds to a pulse length of about $T = 800$ ns. **c** and **d** shows the measured η and P_{FG} with a fixed $\sigma/T = 3.2$ while varying pulse length T. Here we find a maximized gating efficiency $\eta = 0.80$ with the gate pulse length of $T = 960$ ns. The corresponding false gating probability is about $P_{\mathrm{FG}} = 0.04$. The error bar is given by a standard deviation of repeated experiments.

the propagating photon state $\langle(\hat{a}^\dagger)^m\hat{a}^n\rangle$ can be obtained. Based on the moments of photon state and their standard deviations $\delta_{\mathrm{m,n}}$, we can reconstruct the density matrix of the propagating photon mode. We find the most likely density matrix of the propagating photon modes with a maximum likelihood method, by maximizing the log-likelihood function

$$L_{\log} = -\sum_{n,m}\frac{1}{\delta^2_{m,n}}|\langle(\hat{a}^\dagger)^m\hat{a}^n\rangle - \mathrm{Tr}\left[\rho_{\mathrm{ph}}(\hat{a}^\dagger)^m\hat{a}^n\right]|^2, \tag{4}$$

with the physical constraints $\rho_{\mathrm{ph}} \geq 0$ and $\mathrm{Tr}\,\rho_{\mathrm{ph}} = 1$.

In the experiment, based on the already known threshold, the working state of the transistor can be determined in a single shot by measuring the transmitted signal strength from the transistor. For each trial of the measurements, we record the complex amplitudes of the amplified gate photon reflection based on the measured working state of the transistor. To confirm the single-photon switching character of our transistor, we perform quantum state tomography on the conditional gate photon reflections with the method described above. Because a weak gate photon state ($n_{\mathrm{g}} = 0.18$) is used in our experiment, the reflected photon state would be mainly contributed by $|0\rangle$ and $|1\rangle$. Therefore the order of moment we used to reconstruct the photon state is less than 3 ($m + n \leq 2$) and the dimension we used for the reconstructed photon state is also small (up to $|2\rangle$ state), with a sampling number of 5×10^7.

SUPPLEMENTARY NOTE 5 - ERROR ANALYSIS FOR GATE-PHOTON TOMOGRAPHY

As mentioned in the main text, we perform quantum state tomography on the reflected gate photon state conditioned on the signal photon transmission. Here we present a simple error model for the measured photon state, which considers both the photon gating error and the qubit state error. As for the photon gating error, in Supplementary Note3, we measure the photon-gating efficiency η and false gating probability P_{FG} during our photon-gating process. The false gating probability P_{FG} would introduce vacuum state components into the tomography result which is supposed to

be a single-photon state in the ideal case. On the other hand, the non-unity gating efficiency ($\eta < 1$) introduces the Fock state component into the tomography result which is ideally supposed to be a vacuum state. Further, we need to consider the qubit decay/dephasing error during the operation of the transistor, which would also introduce error to the tomography result through the conditioned data acquisition process. Considering these two error sources, the reflected gate photon number conditioned on the measured transmission of signal photons would be

$$\begin{aligned} \langle n_{\mathrm{f}} \rangle &= \frac{P_{\mathrm{nf}}^{\mathrm{T}} \epsilon_{\mathrm{r}}^{\mathrm{nf}} (|\alpha|^2 - \beta) + P_{\mathrm{f}}^{\mathrm{T}} (1 - \epsilon_{\mathrm{r}}^{\mathrm{f}})(1 - P_{\mathrm{FG}})}{P_{\mathrm{nf}}^{\mathrm{T}} \epsilon_{\mathrm{r}}^{\mathrm{nf}} + P_{\mathrm{f}}^{\mathrm{T}} (1 - \epsilon_{\mathrm{r}}^{\mathrm{f}})} \\ \langle n_{\mathrm{nf}} \rangle &= \frac{P_{\mathrm{nf}}^{\mathrm{T}} (1 - \epsilon_{\mathrm{r}}^{\mathrm{nf}})(|\alpha|^2 - \beta) + P_{\mathrm{f}}^{\mathrm{T}} \epsilon_{\mathrm{r}}^{\mathrm{f}} (1 - P_{\mathrm{FG}})}{P_{\mathrm{nf}}^{\mathrm{T}} (1 - \epsilon_{\mathrm{r}}^{\mathrm{nf}}) + P_{\mathrm{f}}^{\mathrm{T}} \epsilon_{\mathrm{r}}^{\mathrm{f}}} \end{aligned} \tag{5}$$

Here $|\alpha|^2 = 0.18$ is the average gate photon number. β is the qubit flip probability when the transistor is gated by the weak coherent state photons, and P_{FG} is the false gating probability, which can be measured as mentioned before. f or nf in subscript and superscript indicates the measurement result when there is a qubit flip or not (or a change of transistor state from the normal-operating mode). For a normally open transistor, f (nf) corresponds to weak (strong) signal photon transmission, while for a normally closed transistor, f (nf) corresponds to strong (weak) signal photon transmission. Therefore in the ideal case $\langle n_{\mathrm{f}} \rangle = 1$ and $\langle n_{\mathrm{nf}} \rangle = 0$.

Considering the photon gating error, when a qubit flip event happens, the expected photon number in the reflected gate pulse reduces from 1 to $1 - P_{\mathrm{FG}}$ due to the non-zero false gating probability. Conditioned on the events without qubit flip, the expected photon number would not be zero but determined by the photon number that does not induce a qubit flip, which can be expressed as $|\alpha|^2 - \beta$.

The qubit state error during the operation of the transistor would lead to a wrong attribute of the qubit flip event, and thus influence the conditional state tomography result. We use $\epsilon_{\mathrm{r}}^{\mathrm{f(nf)}}$ and $P_{\mathrm{f(nf)}}^{\mathrm{T}}$ in Supplementary Equation 5 to take account this error, where $P_{\mathrm{f(nf)}}^{\mathrm{T}}$ represents the qubit flip (or not flip) probability before the signal passing through the transistor, whereas $\epsilon_{\mathrm{r}}^{\mathrm{f(nf)}}$ represents the qubit state error due to the injection of the input signal. $\epsilon_{\mathrm{r}}^{\mathrm{f(nf)}}$ can be directly measured by first preparing the qubit state in $|\mathrm{g}\rangle$ or $|\mathrm{e}\rangle$, and then measuring the signal output from the transistor. Ideally, the transistor would be either switched on or switched off depending on the qubit state. Due to the qubit state error, one would measure a bimodal distribution similar to Supplementary Figure 2. $\epsilon_{\mathrm{r}}^{|\mathrm{g}\rangle(|\mathrm{e}\rangle)}$ can be extracted by comparing the number of histogram counts in the wrong state of operation with the total count number. $\epsilon_{\mathrm{r}}^{|\mathrm{g}\rangle(|\mathrm{f}\rangle)}$ can be also extracted with similar method when operating the transistor in the qubit $\{|\mathrm{g}\rangle, |\mathrm{f}\rangle\}$ subspace. In Supplementary Equation 5, $\epsilon_{\mathrm{r}}^{\mathrm{f}}$ corresponds to $\epsilon_{\mathrm{r}}^{|\mathrm{g}\rangle}$ and $\epsilon_{\mathrm{r}}^{\mathrm{nf}}$ is $\epsilon_{\mathrm{r}}^{|\mathrm{e}\rangle(|\mathrm{f}\rangle)}$ for a normally open transistor, or vice versa for a normally closed transistor.

When the transistor is gated by a photon pulse, the probability of qubit flip we measured would be $P_{\mathrm{f(nf)}}^{M} = P_{\mathrm{f(nf)}}^{\mathrm{T}}(1-\epsilon_{\mathrm{r}}^{\mathrm{f(nf)}}) + P_{\mathrm{nf(f)}}^{\mathrm{T}} \epsilon_{\mathrm{r}}^{\mathrm{nf(f)}}$, by solving these equations, $P_{\mathrm{f(nf)}}^{\mathrm{T}}$ can be obtained. In this way, the conditional tomography result can be analyzed with Supplementary Equation 5. The experimental results and the corresponding theoretical results given by the error model are listed in Supplementary Table 2. One could find that the theoretical results agree with the experimental results, which indicates that the error model presents a plausible description of the switching process.

To have a better understanding about the performance of the transistor, it is worth making a further discussion about the input signal induced qubit flip error $\epsilon_{\mathrm{r}}^{\mathrm{f(nf)}}$. In the experiment, we measure $\epsilon_{\mathrm{r}}^{|\mathrm{g}\rangle} = 0.044$, $\epsilon_{\mathrm{r}}^{|\mathrm{e}\rangle} = 0.075$ for the qubit $\{|\mathrm{g}\rangle, |\mathrm{e}\rangle\}$ subspace with a weak input signal $n_{\mathrm{s}} = 37.2$, and $\epsilon_{\mathrm{r}}^{|\mathrm{g}\rangle} = 0.116$, $\epsilon_{\mathrm{r}}^{|\mathrm{f}\rangle} = 0.167$ for the qubit $\{|\mathrm{g}\rangle, |\mathrm{f}\rangle\}$ subspace with a strong input signal $n_{\mathrm{s}} = 2.62 \times 10^5$. When operating the transistor in qubit $\{|\mathrm{g}\rangle, |\mathrm{e}\rangle\}$ subspace, $\epsilon_{\mathrm{r}}^{|\mathrm{g}\rangle}$ is mainly induced by the thermal population, and $\epsilon_{\mathrm{r}}^{|\mathrm{e}\rangle}$ is induced by the qubit relaxation from $|\mathrm{e}\rangle$ state. It should be pointed out that in $\{|\mathrm{g}\rangle, |\mathrm{f}\rangle\}$ subspace, $\epsilon_{\mathrm{r}}^{|\mathrm{g}\rangle}$ is substantially larger than that in $\{|\mathrm{g}\rangle, |\mathrm{e}\rangle\}$ subspace. This is because of the additional qubit flip error from $|\mathrm{g}\rangle$ to higher excited states induced by the strong input signal [4]. Moreover, $\epsilon_{\mathrm{r}}^{|\mathrm{f}\rangle}$ is also much larger than $\epsilon_{\mathrm{r}}^{|\mathrm{e}\rangle}$. As discussed in the main text, a strong input signal can suppress the cavity non-linearity, which is strongly related with the dispersive shift of the cavity. Considering the limited value of $\chi_{\mathrm{II}}^{\mathrm{gf}}$, it is impossible to promise that the input signal can fully suppress the cavity non-linearity when the qubit is in $|\mathrm{f}\rangle$, whereas without disturbing the cavity non-linearity when the qubit is in $|\mathrm{g}\rangle$ [5, 6]. Therefore there would be still some certain probability that the transistor remains to be switched off when the qubit is in $|\mathrm{f}\rangle$, which induces an addition error in $\epsilon_{\mathrm{r}}^{|\mathrm{f}\rangle}$.

Supplementary Table 2. Average gate photon number, $\langle n_\mathrm{g}\rangle$

Signal photon number and gate phase	State of the transistor	Measured $\langle n_\mathrm{g}\rangle$	Predicted $\langle n_\mathrm{g}\rangle$
$n_\mathrm{s}=37.2$, $\theta=0$	off	0.721	0.725
	on	0.035	0.064
$n_\mathrm{s}=37.2$, $\theta=\pi$	off	0.087	0.069
	on	0.746	0.790
$n_\mathrm{s}=2.62\times10^5$, $\theta=0$	off	0.538	0.578
	on	0.171	0.086
$n_\mathrm{s}=2.62\times10^5$, $\theta=\pi$	off	0.124	0.095
	on	0.634	0.632

SUPPLEMENTARY NOTE 6 - THEORETICAL MODEL FOR PHOTON SWITCHING

A. Gate by an ideal single-photon source

In this section, we present a theoretical description of the single-photon transistor when it is gated by an ideal single-photon source. It is worth mentioning that the theoretical results shown in Fig. 2 of the main text can be derived based on the theoretical model present here.

The photon gating part of the transistor (cavity I and the qubit) can be described by the following Hamiltonian,

$$\hat{H}_\mathrm{qc}=(\omega_\mathrm{c}+\chi)a^\dagger a\left|\mathrm{g}\right\rangle\left\langle\mathrm{g}\right|+(\omega_\mathrm{c}-\chi)a^\dagger a\left|\mathrm{e}\right\rangle\left\langle\mathrm{e}\right|+\omega_\mathrm{q}\left|\mathrm{e}\right\rangle\left\langle\mathrm{e}\right|, \tag{6}$$

where ω_c is the cavity frequency, ω_q is the qubit frequency and χ is the dispersive shift of cavity I. We have written this qubit-cavity Hamiltonian within the dispersive regime. The interaction between flying gate photons and cavity I can be described by following Hamiltonian,

$$\hat{H}_\mathrm{f}=\int k a_k^\dagger a_k dk+\int\sqrt{\frac{\kappa_\mathrm{I}^\mathrm{r}}{2\pi}}(a_k^\dagger a+a_k a^\dagger)dk. \tag{7}$$

$ka_k^\dagger a_k$ in the first term represents photon field with wave-number k, and $\sqrt{\frac{\kappa_\mathrm{I}^\mathrm{r}}{2\pi}}(a_k^\dagger a+a_k a^\dagger)$ in the second term represents the coupling between the propagating photon field and the cavity mode with an out-coupling rate $\kappa_\mathrm{I}^\mathrm{r}$. Thus the whole system is described by Hamiltonian

$$\begin{aligned}\hat{H}_\mathrm{S}=&\hat{H}_\mathrm{qc}+\hat{H}_\mathrm{f}\\=&(\omega_\mathrm{c}+\chi)a^\dagger a\left|\mathrm{g}\right\rangle\left\langle\mathrm{g}\right|+(\omega_\mathrm{c}-\chi)a^\dagger a\left|\mathrm{e}\right\rangle\left\langle\mathrm{e}\right|+\omega_\mathrm{q}\left|\mathrm{e}\right\rangle\left\langle\mathrm{e}\right|+\int k a_k^\dagger a_k dk+\int\sqrt{\frac{\kappa_\mathrm{I}^\mathrm{r}}{2\pi}}(a_k^\dagger a+a_k a^\dagger)dk.\end{aligned} \tag{8}$$

The whole system contains three parts, the superconducting qubit, the photon field in the cavity, and the propagating photon field. For the single-photon gating process, the initial system state can be written as

$$\left|\psi_\mathrm{S}^{(0)}\right\rangle=\frac{1}{\sqrt{2}}(\left|\mathrm{g}\right\rangle+\left|\mathrm{e}\right\rangle)\otimes\left|0\right\rangle\otimes\left|1_\mathrm{p}\right\rangle, \tag{9}$$

The first part represents the state of the superconducting qubit. The second part represents the photon field of the cavity. The last one represents the propagating photon field. The propagating single-photon state can be written as

$$\left|1_\mathrm{p}\right\rangle=\int dt f^*(t)a_t^\dagger\left|0\right\rangle \tag{10}$$

$$=\int d\omega m^*(\omega)a_\omega^\dagger\left|0\right\rangle. \tag{11}$$

$a_t^\dagger$ and $a_\omega^\dagger$ is creation operation of time mode t and frequency mode ω, respectively. $f(t)$ describes the temporal mode of the photon state and $m(\omega)$ describes it in the frequency domain. $f(t)$ and $m(\omega)$ can be converted to each other with Fourier transformation, or

$$f(t)=\frac{1}{\sqrt{2\pi}}\int d\omega m(\omega)e^{i\omega t}, \tag{12}$$

$$m(\omega)=\frac{1}{\sqrt{2\pi}}\int dt f(t)e^{-i\omega t}. \tag{13}$$

$a_k(a_k^\dagger)$ in Supplementary Equation 7 and Supplementary Equation 8 can be replaced by the operator of the photon mode with frequency ω, $a_\omega(a_\omega^\dagger)$, through

$$a_\omega = \frac{1}{\sqrt{v}} a_k, \tag{14}$$

v is the velocity of the propagating photon, where we take $v = 1$ for simplicity. Correspondingly, the single gate photon, $|1_\mathrm{p}\rangle$ with temporal mode $f(t)$ can also be written in the frequency domain with Supplementary Equation 11 and Supplementary Equation 13.

To obtain the time evolution of the system ρ_S during photon gating process, which contains the qubit, the photon field in cavity I and the propagating gate photon, we use the master equation in the Schrödinger picture as

$$\dot{\rho}_\mathrm{S} = -i[\hat{H}_\mathrm{S}, \rho_\mathrm{S}] + \sum_i L_i \rho_\mathrm{S} L_i^\dagger - \frac{1}{2}\{L_i^\dagger L_i, \rho_\mathrm{S}\}. \tag{15}$$

The initial system state ρ_S is given by Supplementary Equation 9, $\rho_\mathrm{S}^{(0)} = \left|\psi_\mathrm{S}^{(0)}\right\rangle \left\langle\psi_\mathrm{S}^{(0)}\right|$. L_i is Lindblad operator which represents the decay or dephasing channel of the system. In our system, $L_1 = \sqrt{\kappa_\mathrm{I}^\mathrm{i}} a$ corresponding to the cavity loss, $L_2 = \sqrt{\gamma_{\mathrm{T}_1}} |\mathrm{g}\rangle \langle\mathrm{e}|$ corresponding to the qubit energy decay and $L_3 = \sqrt{\gamma_\phi}(|\mathrm{g}\rangle\langle\mathrm{g}| - |\mathrm{e}\rangle\langle\mathrm{e}|)$ corresponding to the qubit dephasing are taken into account, where $\gamma_{\mathrm{T}_1} = 1/T_1^\mathrm{ge}$ and $\gamma_\phi = (1/T_2^\mathrm{ge} - 1/2T_1^\mathrm{ge})/2$. For simplicity, the qubit is considered as a two-level system.

By solving Supplementary Equation 15 with the system parameters listed in Supplementary Table 1, the final state after the photon gating process, $\rho_\mathrm{s}^{(\mathrm{f})}$, can be obtained. Therefore, the qubit state flip probability can be obtained as $P_\mathrm{f} = \mathrm{Tr}(|\mathrm{e}\rangle\langle\mathrm{e}| U \rho_\mathrm{s}^{(\mathrm{f})} U^\dagger)$, where $U = \exp(\pi(|\mathrm{g}\rangle\langle\mathrm{e}| - |\mathrm{e}\rangle\langle\mathrm{g}|)/4)$ represents the final $\frac{\pi}{2}$ rotation. This flip probability essentially gives us the gating efficiency by a single photon.

B. Gate by weak coherent state photons

In the experiment, photons in the weak coherent state are used for the photon gating process, instead of an ideal single-photon source. In this case, the system Hamiltonian can be simplified as follow. For a propagating photon mode, it can be described with a real-space operator a_r as

$$a_r = \frac{1}{\sqrt{2\pi}} \int dk \exp(ikr) a_k. \tag{16}$$

Thus the second term in Supplementary Equation 7 can be rewritten as

$$\begin{aligned} \sqrt{\frac{\kappa_\mathrm{I}^\mathrm{r}}{2\pi}} \int (a_k^\dagger a + a_k a^\dagger) dk &= \frac{1}{2\pi}\sqrt{\kappa_\mathrm{I}^\mathrm{r}} \int dr \int dk (\exp(ikr) a_r^\dagger a + \exp(-ikr) a_r a^\dagger) \\ &= \sqrt{\kappa_\mathrm{I}^\mathrm{r}} \int dr (a_r a^\dagger \delta(r) + a_r^\dagger a \delta(r)) \\ &= \sqrt{\kappa_\mathrm{I}^\mathrm{r}} (a_{r=0} a^\dagger + a_{r=0}^\dagger a). \end{aligned} \tag{17}$$

In the above derivation, the definition of Dirac delta function, $\delta(r) = \int \exp(-ikr) dk = \int \exp(ikr) dk$, has been used. $a_{r=0}$ represents the photon field operator at $r = 0$, which is the position of the out-coupling port. In our experiment, the external photon field is a coherent state, thus the operator a_r can be replaced by its mean value, α_r. The gate photons have a Gaussian shaped envelop $f(t) = \exp\left(-\frac{(t-T/2)^2}{2\sigma^2}\right)$, and thus for the out-coupling port the input photon field at time t would be $\alpha_r(t) = \alpha_\mathrm{in} c_0 f(T-t) \exp(-i\omega_\mathrm{c} t)$, where $|c_0|^2 = 1/\int |f(t)^2| dt$ is the normalization factor, α_in is the input gate photon strength with $|\alpha_\mathrm{in}|^2 = n_\mathrm{g}$. The frequency of gate photons is at ω_c. Thus the interaction term (Supplementary Equation 17) can be written as

$$\begin{aligned} \hat{H}_\mathrm{int} &= \sqrt{\kappa_\mathrm{I}^\mathrm{r}} (a_{r=0} a^\dagger + a_{r=0}^\dagger a) = \sqrt{\kappa_\mathrm{I}^\mathrm{r}} (\alpha_r(t) a^\dagger + \alpha_r^*(t) a) \\ &= \frac{\sqrt{2}\sqrt{\kappa_\mathrm{I}^\mathrm{r}}}{\pi^{1/4}\sqrt{\sigma \mathrm{Erf}(T/\sigma)}} \left[\alpha_\mathrm{in} \exp\left(-\frac{(t-T/2)^2}{2\sigma^2}\right) \exp(-i\omega_\mathrm{c} t) a^\dagger + h.c.\right]. \end{aligned} \tag{18}$$

In Supplementary Equation 18, only cavity mode occurs. Thus for simplicity, the propagating photon modes can be traced off from the system and only the qubit and the cavity mode, $\rho_{\rm qc}$, would be taken into consideration. By using the master equation in the Schrödinger picture as

$$\dot{\rho}_{\rm qc} = -i[\hat{H}_{\rm tot}, \rho_{\rm qc}] + \sum_i L_i \rho_{\rm qc} L_i^\dagger - \frac{1}{2}\{L_i^\dagger L_i, \rho_{\rm qc}\}, \tag{19}$$

$\hat{H}_{\rm tot} = \hat{H}_{\rm qc} + \hat{H}_{\rm int}$ is the reduced Hamiltonian. It is worth mentioning that the first term in Supplementary Equation 7 that represents the propagating photon field has been traced off. Lindblad operators for the qubit state, L_2 and L_3, maintain themselves while the cavity decay channel L_1 becomes $L_1 = \sqrt{\kappa_{\rm I}^{\rm r} + \kappa_{\rm I}^{\rm i}}a$. The propagating modes, which have been traced off, become a decay channel of the cavity modes with decay rate $\kappa_{\rm r}$.

By taking qubit state error ϵ_r mentioned in Supplementary Note 5 into account, the event counts we measured when a qubit flip happened would be $N_{\rm m} = N_{\rm tot}P_{\rm f}(1 - \epsilon_{\rm r})$, where $N_{\rm tot}$ is the total event counts. Thus, the dot bars in Fig. 2 of the main text are obtained by adding or subtracting $N_{\rm m}$ from the light blue bars which represent no gate photons.

C. Error estimation for the photon-gating process

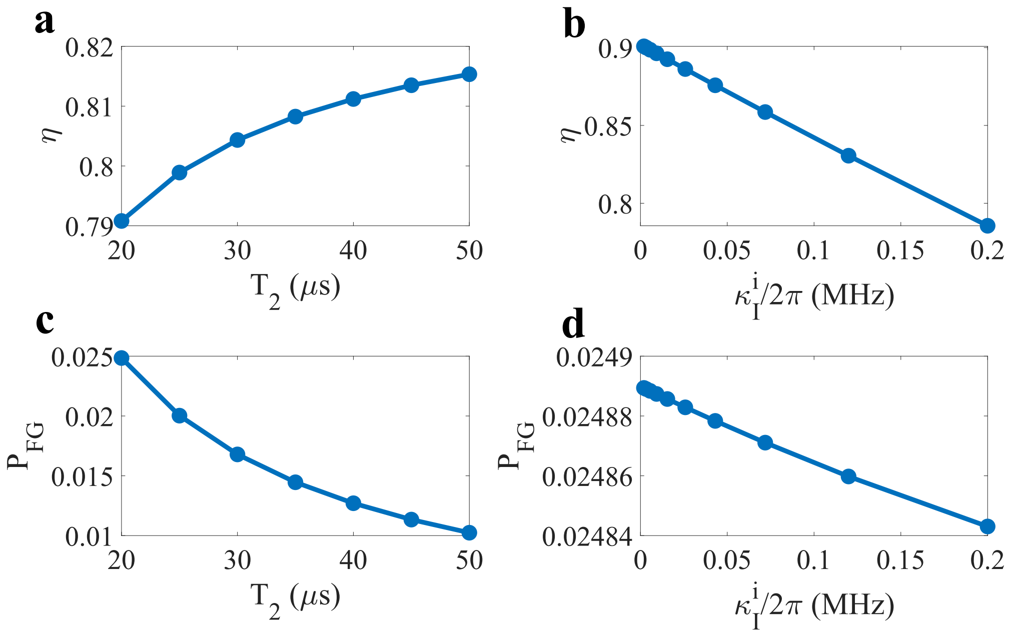

Supplementary Figure 6. An error estimation for the photon-gating process. Based on Supplementary Equation 15, **a** and **c** show the simulation results of the single-photon-gating efficiency η and false gating probability $P_{\rm FG}$ with different qubit dephasing time T_2, where other parameters including the internal loss rate of cavity I are listed in Supplementary Table 1. **b** and **d** show the simulation results of η and $P_{\rm FG}$ with different cavity internal loss $\kappa_{\rm I}^{\rm i}$. The internal loss $\kappa_{\rm I}^{\rm i}/2\pi$ changes from 2 kHz to 0.2 MHz and T_2 equals 20μs. The gate photons have a Gaussian-shaped coherent pulse with width $\sigma = 300$ ns and length $T = 960$ ns.

With the system parameters listed in Supplementary Table 1, the parameters of the gate photons (σ,T,$\alpha_{\rm in}$) and the initial state $\rho_0 = (|{\rm g}\rangle\langle{\rm g}|+|{\rm e}\rangle\langle{\rm e}|+|{\rm g}\rangle\langle{\rm e}|+|{\rm e}\rangle\langle{\rm g}|)\otimes|0\rangle\langle 0|/2$, Supplementary Equation 15 and Supplementary Equation 19 can be solved, and the final state after the photon gating process, $\rho_{\rm f}$, can be obtained. Thus, the corresponding qubit state flip probability can be obtained as $P_{\rm f} = {\rm Tr}(|{\rm e}\rangle\langle{\rm e}|\, U\rho_{\rm f}U^\dagger)$. From the numerical simulation, we obtain an efficiency of $\eta^{\rm theo} = 0.79$ and a false gating probability of $P_{\rm FG}^{\rm theo} = 0.03$, which agrees well with the experimental results shown in Supplementary Figure 4. For the case of the weak coherent state photon gating process, the qubit state flip probability for an average photon number of $n_{\rm g} = 0.18$ is calculated to be $\beta^{\rm theo} = 0.123$, which also agrees well with the experimental result shown in Supplementary Figure 4.

We further present an error budget for the single-photon-gating efficiency η and the false gating probability $P_{\rm FG}$ based on the theoretical model. We consider the influence of internal loss of cavity I and the limited qubit coherence

on the performance of the photon-gating process. In Supplementary Figure 6, we calculate η and P_{FG} for different qubit dephasing time T_2 and cavity internal loss rate $\kappa_{\mathrm{I}}^{\mathrm{i}}$. From Supplementary Figure 6**b**, a smaller cavity internal loss rate is preferred for a larger single-photon gating efficiency η. If the cavity internal loss rate could be reduced to a state-of-the-art value of 2 kHz [7], η can be improved to about 90%, with a moderate qubit coherence time of 20 μs. On the other hand, from Supplementary Figure 6**c**, an improved qubit coherence is helpful in reducing the false gating probability P_{FG}, which would be about 1% by improving T_2 to 50 μs.

If we take the state-of-art system parameters of qubit dephasing time $T_2 = 50\mu$s and $\kappa_{\mathrm{I}}^{\mathrm{i}} = 2$ kHz, the photon-gating process is predicted to have a gating efficiency $\eta^{\mathrm{sim}} = 0.93$ and false gating probability $P_{\mathrm{FG}}^{\mathrm{sim}} = 0.01$. We note that in this case, the gating efficiency could be further improved by optimizing the shape of the gate photon pulse, considering that in the simulation we use the experimentally optimized pulse (Gaussian-shaped, with width $\sigma = 300$ ns and length T = 960 ns) for a total cavity linewidth of 2 MHz. If we take $\kappa_{\mathrm{I}}^{\mathrm{i}} = 2$ kHz, the total linewidth of cavity I would be about 1.8 MHz, which in principle requires a longer gate photon pulse to as an effective feeding to cavity I.

SUPPLEMENTARY NOTE 7 - VARIED SIGNAL PULSE LENGTH

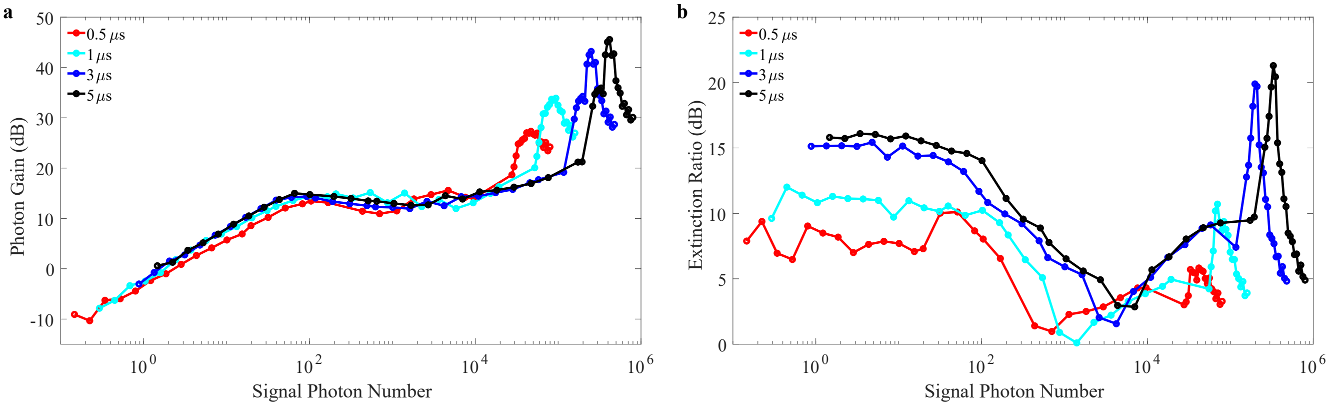

Supplementary Figure 7. Gain and extinction ratio for different signal photon pulse lengths. **a** and **b** show the extracted gain and extinction ratio of our device, respectively, when the transistor is fed with varied signal photons with different pulse lengths ($5\mu s$, $3\mu s$, $1\mu s$, $0.5\mu s$). The photon number changes from 0.14 to 0.8×10^6. The transistor is operated in the qubit $\{|\mathrm{g}\rangle, |\mathrm{e}\rangle\}$ subspace. Note that since we pick up the best measured gain and extinction ratio for each of the input signal strength, the signal frequency is not necessarily kept unchanged.

In the main text, we show the gain and extinction ratio of our single-photon transistor with a signal pulse length of 10 μs. In this part, we discuss the performance of the single-photon transistor with varying lengths of the signal pulse. Supplementary Figure 7 shows the measured gain and extinction ratio for signal pulse length of 5 μs, 3 μs, 1 μs and 0.5 μs in the qubit $\{|\mathrm{g}\rangle, |\mathrm{e}\rangle\}$ subspace.

For signal photon lengths of 5 μs and 3 μs, the transistor generally shows similar performance as that with the 10 μs signal pulse presented in the main text. One could see a linearly increased photon gain as a function of input photon number and an average extinction ratio above 15 dB when the signal contains less than 100 photons. When the signal strength is strong enough to remove the non-linearity of the qubit-coupled cavity, one could see a peak of gain and extinction ratio in this regime. The peaks appear at different photon numbers for different lengths of signal pulse, which is not surprising considering that the peak position is determined by the cavity photon number, which is further determined by the power of input signal photons, but not the signal photon number.

For signal photon lengths of 1 μs and 0.5 μs, the transistor shows a slightly lower gain but degraded extinction ratio when the input signal contains less than 100 photons. The peak values of gain and extinction ratio also show clear decay when the transistor is fed with a strong input signal. From Supplementary Table 1, we find that the total linewidth of cavity II, $\kappa_{\mathrm{II}}^{\mathrm{tot}} = \kappa_{\mathrm{II}}^{\mathrm{i}} + \kappa_{\mathrm{II}}^{\mathrm{in}} + \kappa_{\mathrm{II}}^{\mathrm{out}} = 2\pi \times 0.3\ \mu s^{-1}$, thus it has a response time of about $\tau = 1/\kappa_{\mathrm{II}}^{\mathrm{tot}} \approx 0.5\ \mu s$. For signal photons with a pulse length of 3 μs ($\approx 6\tau$), 5 μs ($\approx 10\tau$) and 10 μs ($\approx 20\tau$), they are long enough compared to the cavity response, and thus can well-fitted into the bandwidth of cavity II. While for shorter signal pulses, such as 1 μs ($\approx 2\tau$) and 0.5 μs ($\approx \tau$), the bandwidth of the signal pulse is comparable with or even small than that of cavity II. The input signal cannot be effectively fed into cavity II, which leads to the degraded extinction ratio and

gain.

.

SUPPLEMENTARY NOTE 8 - CAVITY TRANSMISSION IN THE PRESENCE OF A STRONG INPUT SIGNAL

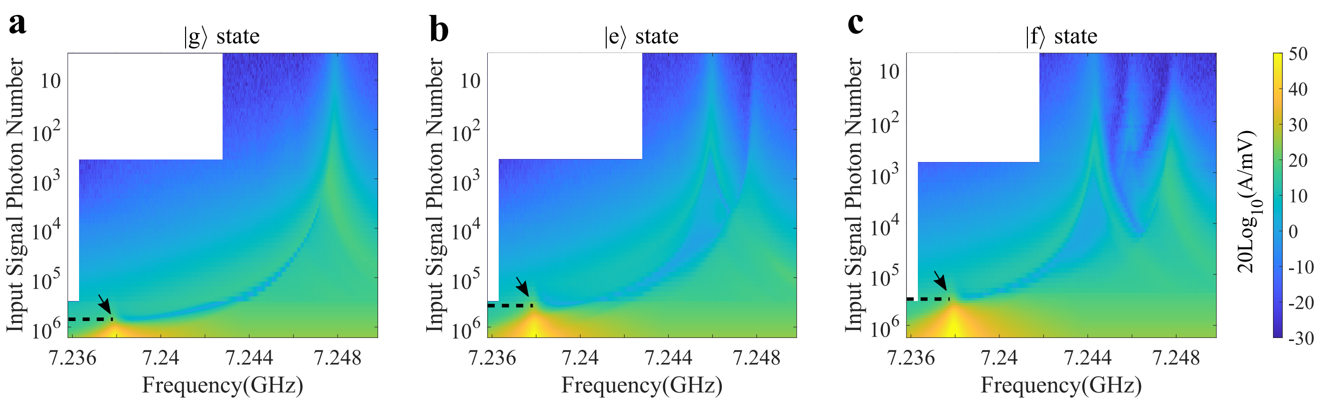

Supplementary Figure 8. The measured transmission of cavity II with varied input signal strength. **a**, **b**, and **c** show measured cavity transmission (in logarithmic scale) for qubit in the state of $|g\rangle$, $|e\rangle$ and $|f\rangle$, respectively. When the input signal photon number is less than 100, the measured transmission originates from the qubit-state-dressed cavity mode, whose frequency shows clear qubit state dependence. The transmission spectra for the qubit state in $|e\rangle$ and $|f\rangle$ show more than one peak due to the qubit relaxation during the measurement. With an increasing signal photon number, the dressed-cavity-mode-related transmission gets suppressed, until the bright mode appears at around 7.23795 GHz (indicated by the black arrows), which is the bare frequency of cavity II. The critical signal strength that results in the bright mode shows clear qubit state dependence.

As described in the main text, our single-photon transistor can operate at a very large input signal strength, thus yielding a large value of gain. In this part, we provide more data to explain the details of this high-power regime.

In our transistor design, cavity II is dispersively coupled to the qubit, which enables the switch operation thanks to the qubit-state-dependent transmission. Considering the intrinsic non-linearity of the qubit-state-dressed cavity mode, the photon population in the cavity would shift its resonance frequency. Therefore, it is generally believed that the cavity mode would get 'blurred' when it is fed with a strong signal, known as the photon-blockade effect. Supplementary Figure 8**a** shows the steady-state cavity transmission when the qubit is in $|g\rangle$, from which one could see that the cavity transmission is suppressed when the input signal contains more than 100 photons. It results in the flat gain and dropped extinction ratio in the medium power regime in Fig. 3 of the main text.

When further increasing the signal power, it has been reported that when the photon number in the cavity exceeds a certain threshold, the non-linearity of the dressed cavity mode can be suppressed and the high-contrast transmission of the linear cavity mode can be recovered [5, 6, 8]. As shown in Supplementary Figure 8**a**, for the qubit state in $|g\rangle$, when the input signal strength exceeds 5×10^5 photons, the cavity transmission is recovered with a relatively red-shifted frequency of about 7.23795 GHz, which is the bare frequency of cavity II.

Moreover, the critical input signal strength that triggers the transition shows a strong dependence on the state of the qubit. This can be understood because the resonance frequency of the linear cavity mode is detuned from that of the qubit-cavity hybrid mode, and the detuning is varied with different qubit states due to the dispersive interaction between the qubit and the cavity. Therefore, to populate the cavity to the critical photon number, the required input signal strength would be different for different qubit states. Supplementary Figure 8**b** and Supplementary Figure 8**c** show the measured cavity transmission when the qubit is in $|e\rangle$ and $|f\rangle$. Compared with the result shown in Supplementary Figure 8**a**, weaker signal strength is required to suppress the cavity non-linearity when the qubit is in $|e\rangle$ and $|f\rangle$. Intuitively, a larger dispersive shift would yield a larger difference in the critical signal strength, which could result in a higher gain and a broader peak of the high-gain region for the single-photon transistor, as shown in Fig. 3 of the main text.

SUPPLEMENTARY REFERENCES

[1] Kono, S., Koshino, K., Tabuchi, Y., Noguchi, A. and Nakamura, Y. Quantum non-demolition detection of an itinerant microwave photon. *Nature Physics*. **14**, 546-549 (2018)

[2] Eichler, C., Bozyigit, D. and Wallraff, A. Characterizing quantum microwave radiation and its entanglement with superconducting qubits using linear detectors. *Phys. Rev. A*. **86**, 032106 (2012,9)

[3] Wang, Z., Bao, Z., Wu, Y., Li, Y., Cai, W., Wang, W., Ma, Y., Cai, T., Han, X., Wang, J., Song, Y., Sun, L., Zhang, H. and Luming Duan A flying Schrödinger's cat in multipartite entangled states. *Science Advances*. **8**, eabn1778 (2022)

[4] Slichter, D., Vijay, R., Weber, S., Boutin, S., Boissonneault, M., Gambetta, J., Blais, A. and Siddiqi, I. Measurement-Induced Qubit State Mixing in Circuit QED from Up-Converted Dephasing Noise. *Phys. Rev. Lett.*. **109**, 153601 (2012,10)

[5] Bishop, L., Ginossar, E. and Girvin, S. Response of the Strongly Driven Jaynes-Cummings Oscillator. *Phys. Rev. Lett.*. **105**, 100505 (2010,9)

[6] Reed, M., DiCarlo, L., Johnson, B., Sun, L., Schuster, D., Frunzio, L. and Schoelkopf, R. High-Fidelity Readout in Circuit Quantum Electrodynamics Using the Jaynes-Cummings Nonlinearity. *Phys. Rev. Lett.*. **105**, 173601 (2010,10)

[7] Reagor, M., Pfaff, W., Axline, C., Heeres, R., Ofek, N., Sliwa, K., Holland, E., Wang, C., Blumoff, J., Chou, K., Hatridge, M., Frunzio, L., Devoret, M., Jiang, L. and Schoelkopf, R. Quantum memory with millisecond coherence in circuit QED. *Phys. Rev. B*. **94**, 014506 (2016,7)

[8] Boissonneault, M., Gambetta, J. and Blais, A. Improved Superconducting Qubit Readout by Qubit-Induced Nonlinearities. *Phys. Rev. Lett.*. **105**, 100504 (2010,9)
